# MicroRNAs in renal fibrosis

**DOI:** 10.3389/fphys.2015.00050

**Published:** 2015-02-20

**Authors:** Arthur C.-K. Chung, Hui Y. Lan

**Affiliations:** ^1^Partner State Key Laboratory of Environmental and Biological Analysis, Department of Chemistry, Hong Kong Baptist UniversityHong Kong, China; ^2^HKBU Institute for Research and Continuing EducationShenzhen, China; ^3^Department of Medicine and Therapeutics, Li Ka Shing Institute of Health Sciences, Chinese University of Hong KongHong Kong, China

**Keywords:** microRNAs, kidney diseases, renal fibrosis, TGF-β signaling, biomarkers

## Abstract

MicroRNAs (miRNAs) are endogenous short non-coding RNAs that regulate most of important cellular processes by inhibiting gene expression through the post-transcriptional repression of their target mRNAs. In kidneys, miRNAs have been associated in renal development, homeostasis, and physiological functions. Results from clinical and experimental animal studies demonstrate that miRNAs play essential roles in the pathogenesis of various renal diseases. Chronic kidney diseases (CKD) is characterized by renal fibrosis. Transforming growth factor beta (TGF-β) is recognized as a major mediator of renal fibrosis because it is able to stimulate the accumulation of extracellular matrix (ECM) proteins to impair normal kidney function. Recently, emerging evidence demonstrate the relationship between TGF-β signaling and miRNAs expression during renal diseases. TGF-β regulates expression of several microRNAs, such as miR-21, miR-192, miR-200, miR-433, and miR-29. MiR-21, miR-192, and miR-433 which are positively induced by TGF-β signaling play a pathological role in kidney diseases. In contrast, members in both miR-29 and miR-200 families which are inhibited by TGF-β signaling protect kidneys from renal fibrosis by suppressing the deposition of ECM and preventing epithelial-to-mesenchymal transition, respectively. Clinically, the presence of miRNAs in blood and urine has been examined to be early biomarkers for detecting renal diseases. From experimental animal studies of CKD, targeting microRNAs also provides evidence about therapeutic potential of miRNAs during renal diseases. Now, it comes to the stage to examine the exact mechanisms of miRNAs during the initiation and progression of renal diseases. Therefore, determining the function of miRNAs in renal fibrosis may facilitate the development of both early diagnosis and treatment of renal diseases.

## Introduction

MicroRNAs (miRNAs) are small, endogenous, non-coding RNAs that regulate various cellular processes such as death, differentiation, proliferation, metabolism, and pathophysiology of many diseases via the regulation of target gene expression. During recent decades, the understandings of miRNAs in molecular mechanisms on various disease processes are expanding rapidly. In the kidney diseases, miRNAs also play a key role in renal fibrosis.

MiRNAs bind to their respective target mRNAs and recruit the RNA-induced silencing complex (RISC). There are multiple steps of the biogenesis of miRNAs. Firstly, after having transcribed by RNA polymerase II or RNA polymerase III as long stem-loop primary miRNA (Pri-miR) in the nucleus, the Pri-miR is then cleaved into a double-stranded shorter miRNA precursor (Pre-miR) by RNase III enzyme Drosha and its partner DGCR8 (DiGeorge syndrome critical region 8) (Lee et al., 2003; Gregory et al., 2004). These Pre-miR will next be exported into the cytoplasm by the Ran-GTP and Exportin-5 (Du and Zamore, 2005). Pre-miR is further cleaved into the mature form, a 20–22 base pairs (bp) double-stranded RNA, in the cytoplasm by another RNase III enzyme Dicer. This mature miRNA-miRNA duplex is unwound and the functional strand (“guide strand”) is loaded onto the RISC (Filipowicz, 2005). The mature miRNA induces the RISC complex to bind to the 3′ untranslated region (3′ UTR) of a target messenger RNA (mRNA). This will result in post-transcriptional gene silencing by mRNA degradation or by translation inhibition. Therefore, miRNAs is able to suppress target gene expression by mRNA degradation, translation inhibition or transcriptional inhibition.

Early studies by microarray assays demonstrate that the abundance of miR-192, -194, -204, -215, and -216 are high in the kidney when compared with other organs (Sun et al., 2004; Tian et al., 2008). These studies suggest the potential role of miRNAs in kidney function. So far, more and more miRNAs have been described in the development, hemostasis and diseases in the kidney (Kato et al., 2009; Li et al., 2010; Bhatt et al., 2011; Lorenzen et al., 2011). As many comprehensive reviews about the biogenesis of miRNAs and the role of miRNAs in normal kidney have been published (Du and Zamore, 2005; Filipowicz, 2005; Saal and Harvey, 2009; Wessely et al., 2010; Bhatt et al., 2011; Lorenzen et al., 2011; Chung et al., 2013b; Li et al., 2014), this review will focus on recent novel findings into the implications for miRNAs in renal fibrosis.

## Role of TGF-β signaling in renal fibrosis

**Figure 1:**
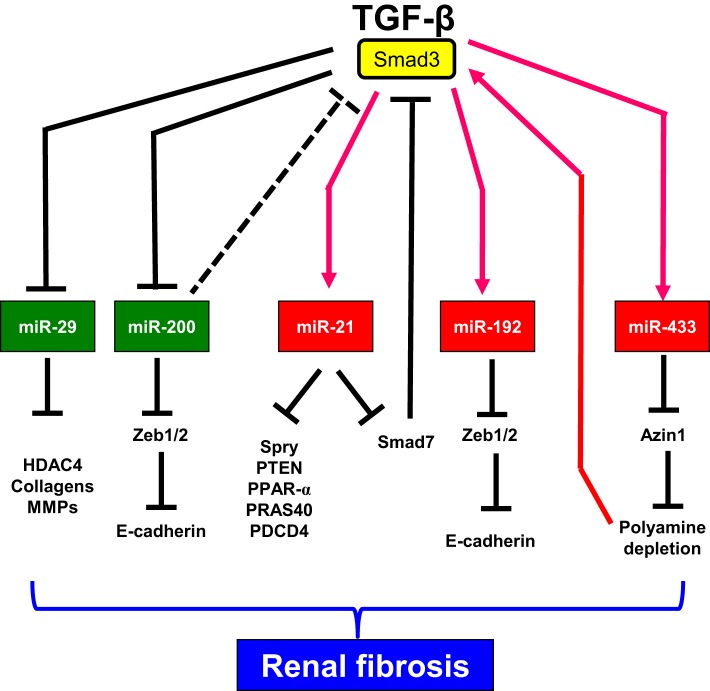
**Mechanisms of TGF-β-regulated miRNAs in renal fibrosis**. TGF-β promotes fibrosis by inducing miR-21, miR-433, and miR-192 but suppressing miR-29 and miR-200 during renal injury. miR-192, miR-433, and miR-21 play a pathological role in kidney fibrosis through a feed-forward loop to amplify TGF-β signaling and promote fibrosis. In contrast, members in miR-29 and miR-200 families play a protective role in renal fibrosis by inhibiting the deposition of extracellular matrix and preventing epithelial-to-mesenchymal transition (EMT), respectively.

Renal fibrosis is the common feature of chronic kidney disease (CKD) progressing to end-stage renal failure. Renal fibrosis is generally characterized either by interstitial extracellular matrix (ECM), or myofibroblast accumulation, and destruction of renal tubules (Bottinger, 2007; Liu, 2011). Transforming growth factor-beta (TGF-β) is the well-known master cytokine/growth factor in fibrosis (Roberts, 1998; Wang et al., 2005; Meng et al., 2010, 2012a,b; Lan and Chung, 2011) (Figure 1). Smad2 and Smad3 are the important downstream mediators of TGF-β signaling (Massague and Chen, 2000; Miyazono, 2000; Chung et al., 2010b; Zhou et al., 2010a; Chen et al., 2011; Li et al., 2013b; Meng et al., 2013). During fibrosis, TGF-β is capable of inducing many fibrogenic genes, such as ECM proteins, via Smad2, Smad3, or mitogen-activated protein kinases (MAPKs) (Hoffman et al., 1998; Schnaper et al., 2003; Chung et al., 2009). Recent studies show that TGF-β also regulates several miRNAs during renal fibrosis. TGF-β1 induces miR-21, miR-192, miR-491-5p, miR-382, miR-377, miR-214, and miR-433, but suppresses the miR-29 and miR-200 families (Kriegel et al., 2010, 2012a; Kantharidis et al., 2011; Lan and Chung, 2012; Chung et al., 2013a) (Table 1). All these TGF-β-regulated miRNAs have been shown to participate in the events during renal fibrosis (Figure 1). Furthermore, expression of these miRNAs is altered when the kidneys are injured in the experimental mouse models of renal injury (Kantharidis et al., 2011; Lan and Chung, 2012; Chung et al., 2013a), suggesting that they play essential roles in TGF-β-induced fibrosis. In this review, we will discuss the recent findings of five groups of TGF-β-regulated miRNAs, including miR-21, miR-29, miR-192, miR-200, and miR-433 because they have been shown to modulate TGF-β-induced renal fibrosis. MiR-21, miR-192, and miR-433 promote fibrosis while miR-29 and miR-200 families inhibit fibrosis.

**Table 1:** **Roles of microRNAs in animal models of kidney diseases**.

**microRNA**	**Mouse model of kidney diseases**	**Pathological output**	**References**
**miRNAs WHICH ARE INDUCED BY TGF-β**			
miR-21	UUO, ISI, DN in *db/db* mice	Fibrosis and inflammation	Zhong et al., 2011, 2013; Chau et al., 2012
miR-192	UUO, STZ induced DN, DN in *db/db* mice	Fibrosis and EMT	Kato et al., 2007; Chung et al., 2010a; Putta et al., 2012
miR-216a	STZ induced DN, DN in db/db mice	Increases col1a2 expression	Kato et al., 2010
miR-377	Spontaneous and STZ induced DN	Fibronectin expression	Wang et al., 2008
miR-491-5p	UUO (rat)	Induces Par-3 degradation	Zhou et al., 2010b
miR-382	UUO	Suppresses E-cadherin	Kriegel et al., 2012a
miR-433	UUO	Fibrosis, polyamine depletion	Li et al., 2013a
miR-29a,b,c	UUO, DN in *db/db* mice	Fibrosis	Du et al., 2010; Qin et al., 2011; Chen et al., 2014; Lin et al., 2014
miR-200a,b,c, miR-141, miR-429	UUO	EMT	Oba et al., 2010; Wang et al., 2011; Xiong et al., 2012
	STZ induced DN, adenine-induced		

*EMT, epithelial-to-mesenchymal transition; DN, diabetic nephropathy; ISIS, ischemia reperfusion injury; STZ, streptozotocin; UUO, Unilateral Ureteral Obstruction*.

## microRNAs in TGF-β-induced renal fibrosis

### miR-29

Three members of the miR-29 family are encoded from two distinct genomic loci in both human and rodent genomes (Kriegel et al., 2012b). All miR-29 members have the identical seed binding sequence and they all bind to the identical set of target genes (Kriegel et al., 2012b). Findings from clinical studies and experimental models suggest the anti-fibrotic effects of miR-29. Abundance of miR-29s is always high in the kidney, lung, and heart (Kriegel et al., 2012b). However, their abundance is greatly reduced in animal models and human samples of fibrotic diseases in heart, lung, and kidney (van Rooij et al., 2008; Qin et al., 2011; Xiao et al., 2012; Zhang et al., 2014).

By employing microarrays and real-time PCR assays in the mouse model of unilateral ureteral obstruction (UUO), abundance of miR-29a, -29b, and -29c is substantially decreased in the fibrotic kidney of UUO wild-type mice but significantly induced in Smad3 knockout (KO) mice in which renal fibrosis was inhibited (Qin et al., 2011). This negative relationship of miR-29 in TGF-β-dependent fibrosis is further confirmed by the reduced expression of miR-29a and miR-29b in renal TECs, lung, and cardiac fibroblasts after treatment of TGF-β1 (van Rooij et al., 2008; Qin et al., 2011; Xiao et al., 2012).

Consistent with the results in heart that suppression of miR-29b increases the levels of fibrotic markers (van Rooij et al., 2008; Ye et al., 2010; Zhang et al., 2014), overexpression of miR-29 reduces but inhibition of miR-29 enhances abundance of fibrotic markers in mouse embryonic fibroblasts (MEF) and TECs under diabetic condition, salt-induced hypertensive conditions, or after TGF-β treatment, confirming the anti-fibrotic influence of miR-29 (Du et al., 2010; Liu et al., 2010b; Qin et al., 2011; Chen et al., 2014) (Figure 1). These reults are further confirmed by mouse models of unilateral ureteral obstruction and diabetic nephropathies (Qin et al., 2011; Chen et al., 2014). Delivery of *miR-29b* gene either before or after established obstructive and diabetic nephropathies effectively suppresses the progression of renal fibrosis. The ability to inhibit TGF-β-mediated deposition of ECM by miR-29 may be mechanism of how miR-29 protects kidney from fibrosis because more than 20 different ECM-related genes are predicted to be miR-29 targets and some of them are positively regulated by TGF-β signaling (van Rooij et al., 2008). A recent study demonstrates that increasing miR-29a action also protects against diabetic podocytopathy by suppressing HDAC4 signaling, nephrin ubiquitination, and urinary nephrin excretion associated with diabetes and restoring nephrin acetylation (Lin et al., 2014). In conclusion, miR-29 is a downstream inhibitor of TGF-β-mediated fibrosis and may have therapeutic potential for diseases involving fibrosis.

### miR-200

The miR-200 family includes miR-200a, -200b, -200c, -429, and -141. This family is known to maintain epithelial differentiation (Howe et al., 2012) because they were firstly discovered by their ability to restore an epithelial phenotype in breast cancer cell lines by inhibiting ZEB1 and ZEB2, the E-cadherin transcriptional repressors (Burk et al., 2008; Gregory et al., 2008; Korpal et al., 2008; Park et al., 2008) (Figure 1). During obstructive and diabetic nephropathy, abundance of miR-200a and miR-141 are reduced in the fibrotic kidneys (Wang et al., 2011; Xiong et al., 2012). These findings are confirmed by *in vitro* studies that abundance of the miR-200 family in TECs is reduced in a TGF-β/Smad-dependent manner (Wang et al., 2011; Xiong et al., 2012). However, opposite results are shown in another study that renal expression of miR-200s is elevated in the mouse model of UUO (Oba et al., 2010). However, the differences of miR-200 expression in these mouse models of kidney diseases are possibly due to the differences in the origin of cell lines examined, the treatments performed, and the use of different animal models between studies. Side by side comparison of these mouse models and cell culture studies should be performed to understand the exact mechanism. In spite of the difference of miR-200 expression in fibrotic kidneys, the anti-fibrotic role of miR-200 family is confirmed by gene delivery of miR-200b in fibrotic kidney. A single injection of miR-200b precursor is sufficient to inhibit the up-regulation of collagens and fibronectin in obstructed kidneys (Oba et al., 2010).

### miR-21

MiR-21 is one of the first microRNAs to be described as an oncomir because it is associated in the genesis and progression of human cancers (Jazbutyte and Thum, 2010). MiR-21 expression is closely related to fibrosis and it is up-regulated by TGF-β1 (Zavadil et al., 2007; Davis et al., 2008, 2010). The first report of miR-21 in fibrosis is firstly shown in heart failure (Thum et al., 2008). Its expression is induced in cardiac fibroblasts of the failing hearts and delivery of miR-21 antagomir into a mouse model of cardiac hypertrophy inhibits interstitial fibrosis and restores the cardiac function (Thum et al., 2008). Similarly, elevation of miR-21 expression is found in the patients with idiopathic pulmonary fibrosis and in mice with bleomycin-induced lung fibrosis (Liu et al., 2010a). Suppressing miR-21 by antisense oligonucleotides inhibits lung fibrosis in mice (Liu et al., 2010a).

Although the abundance of miR-21 is low in normal kidneys, its abundance is greatly increased in both patient samples of kidney diseases and animal models of CKD and acute kidney injury (AKI) (Godwin et al., 2010; Zhong et al., 2011, 2013; Chau et al., 2012; Xu et al., 2012; Wang et al., 2013). From the studies in mouse models of obstructive and diabetic nephropathy, high abundance of miR-21 is observed in both tubulointerstitial and glomerular area where fibrosis happens (Zhong et al., 2011, 2013; Wang et al., 2013). In another study of ischemia-reperfusion injury, similar elevation of renal miR-21 is also observed (Godwin et al., 2010; Xu et al., 2012). These results suggest the pathological role of miR-21 in renal diseases.

Similarly, miR-21 positively regulates expression of ECM and α-SMA in tubular epithelial cell (TEC) and mesangial cells (MCs) after treatment of TGF-β1 or under diabetic condition (Zhong et al., 2011, 2013). In addition, knockdown of miR-21 inhibits but overexpression of miR-21 in kidney cells enhances renal fibrosis under diabetic condition or after treatment with TGF-β1 (Zarjou et al., 2011; Zhong et al., 2011, 2013).

Targeting miR-21 should possess a therapeutic potential to ameliorate the disease related to fibrosis because inhibition of miR-21 is effectively to decrease fibrosis in rodent models of heart, lung, and kidney diseases (Thum et al., 2008; Liu et al., 2010a; Zhong et al., 2011). In the mouse models of diabetic and obstructive nephropathy, inhibition of miR-21 also improves kidney function and halts the progression of renal injury (Zhong et al., 2011, 2013).

The results from miR-21 KO mice further confirm the pathological role of renal fibrosis (Chau et al., 2012). Consistence with the results of unilateral ureteral obstruction (UUO) and renal ischemia reperfusion injury (ISI) models, *miR-21* gene deficiency in mice reduces renal fibrosis, tubule atrophy, and P42/P44 MAP kinase pathway activation in diseased kidneys when compared with wild type mice (Chau et al., 2012). Furthermore, a negative relationship between the presence of miR-21 and genes which are involved in lipid metabolism, fatty acid oxidation, and redox regulation is found in miR-21 KO kidneys. More interestingly, this study also demonstrate that suppression of peroxisome proliferator-activated receptor-α (PRAR-α) by miR-21 may be one of mechanisms of how miR-21 promotes renal fibrosis by (Figure 1) (Chau et al., 2012).

The investigation of how miR-21 regulates fibrosis is still ongoing. Results from the studies in cardiac fibrosis show that *Phosphatase and tensin homolog* (*PTEN*) and *Sprouty* (*SPRY*) are two of potential targets of miR-21 (Thum et al., 2008; Roy et al., 2009) (Figure 1). It is found that miR-21 can suppress PTEN to activate phosphatidylinositide 3-kinases (PI3K) and Akt activity, and next increases MMP-2 abundance (Roy et al., 2009). As SPRY is an inhibitor of Ras/MEK/ERK, suppression of SPRY by miR-21 will activate ERK to promote TGF-β-induce fibrosis (Ding et al., 2008). In heart, suppression of miR-21 reduces ERK-MAPK activity and interstitial fibrosis (Thum et al., 2008). Recent study on diabetic nephropathy shows that *Smad7* and AKT1 substrate 1 (*PRAS40*), a negative regulator of Tor complex 1 (TORC1), are potential targets of miR-21 (Dey et al., 2011; Zhong et al., 2013). Negative correlation between miR-21 and *proinflammatory programmed cell death 4* (*PDCD4*) has also been reported in TEC with induction of ischemia (Godwin et al., 2010). As more and more miR-21 target genes which are related to fibrosis are found, further studies should be done to clarify how miR-21 exactly controls these target genes during renal fibrosis.

### miR-192

As mentioned above, abundance of miR-192 is high in the normal kidney, as compared with other organs (Sun et al., 2004; Tian et al., 2008). Pro-fibrotic role of miR-192 has been report in several studies from rodent models of kidney diseases, and in both MCs and TECs (Kato et al., 2007; Chung et al., 2010a; Putta et al., 2012). High abundance of miR-192 is detected in glomeruli isolated from diabetic mice (Kato et al., 2007). Treatment with TGF-β or high glucose in MCs and TEC up-regulates miR-192 expression (Kato et al., 2007; Chung et al., 2010a). Suppression of zinc finger E-box binding homeobox 1/2 (Zeb1/2) expression by miR-192 may be one of the mechanism of how miR-192 regulates TGF-β-induced collagen expression in MCs (Kato et al., 2007) (Figure 1). Similarly, inhibition of miR-192 reduces but overexpression of miR-192 enhances TGF-β1-induced collagen accumulation in TEC (Chung et al., 2010a). These results are further confirmed by a study in a mouse model of type I diabetes. An *in vivo* inhibition of renal miR-192 greatly up-regulates renal expression of Zeb1/2 and inhibits proteinuria, and expression of collagen and fibronectin (Putta et al., 2012). The pathological role of miR-192 in diabetic nephropathy is then supported by the findings in miR-192 KO mice (Deshpande et al., 2013). Deletion of *miR-192* gene in type I diabetic mice reduced albuminuria, proteinuria, renal fibrosis, and hypertrophy as compared to diabetic wild-type mice (Deshpande et al., 2013). Taken together, these studies demonstrate a pro-fibrotic role of miR-192 in TGF-β-dependent renal fibrosis observed in animal models of diabetic and obstructive nephropathy (Kato et al., 2007; Chung et al., 2010a; Putta et al., 2012).

However, the reverse is true in human nephropathy (Krupa et al., 2010; Wang et al., 2010). Remarkably, reduction of miR-192 expression is observed in human TECs after TGF-β1 treatment or in the human diseased kidneys (Krupa et al., 2010; Wang et al., 2010). This reduction of miR-192 expression correlates with tubulointerstitial fibrosis and a low GFR in diabetic patients. These significant differences in miR-192 expression in human and animal models of diabetic nephropathy requires further investigation to identify role and mechanism of miR-192's action during renal fibrosis in different species.

### miR-433

Early studies of miR-433 focus on its role in cancer (Jung et al., 2000; Luo et al., 2009). Recently, miR-433 has been found to be one of the important components of TGF-β/Smad3 driven renal fibrosis (Li et al., 2013a). Similar to miR-21 and miR-192, renal miR-433 expression is induced after UUO. *In vitro*, TGF-β promotes fibrosis in TEC by inducing miR-433 expression (Li et al., 2013a). This induction requires the activation of TGF-β signaling. In addition, inhibition of miR-433 suppresses but overexpression of miR-433 enhances TGF-β1-induced collagen matrix accumulation in TEC (Li et al., 2013a). More importantly, suppression of miR-433 *in vivo* reduces renal fibrosis and halts the progression of renal fibrosis in established obstructive nephropathy. Antizyme inhibitor 1 (Azin1), a protective protein in kidney fibrosis, is found to be a target of miR-433 that overexpression of Azin1 is able to suppress expression of fibrotic proteins in TEC (Li et al., 2013a). As Azin1 promotes polyamine synthesis and polyamine depletion can activate TGF-β signaling by increasing the expression levels of TGF-β1, TβRI, and Smad3 (Patel et al., 1998; Liu et al., 2003), elevation of miR-433 during renal fibrosis forms a positive feedback loop to amplify TGF-β signaling by suppressing Azin1 expression.

## Regulation of fibrosis-related microRNAs by TGF-β signaling

Although the precise mechanism of how TGF-β signaling regulates miRNA expression during renal fibrosis is still continuing, recent evidence demonstrates that TGF-β signaling induces the synthesis of fibrosis-related microRNAs either by increasing transcription, or by enhancing posttranscriptional processing of primary miRNA transcript. Davis et al demonstrate that TGF-β signaling enhances the processing of primary transcripts of some microRNAs into its active form by the Drosha complex, such as miR-21 (Davis et al., 2008). Smad3, as one of the receptor-Smads, physically interacts with the Drosha complex to stimulate the production of mature miR-21 from the pri-miR-21 transcript (Figure 1). In addition, a consensus sequence (R-SBE) is found to be located within the stem region of the primary transcripts of TGF-β-regulated-miRs (pri-T-miRs) (Davis et al., 2010). The direct binding between Smads and the R-SBE will initialize the TGF-β-induced recruitment of Drosha, and DGCR8 to pri-T-miRs and enhance the processing of pri-T-miRs (Davis et al., 2010).

Our laboratory demonstrates that TGF-β/Smad3 signaling is able to regulate the transcription of miR-21, miR-192, miR-433, and the miR-29 family during renal diseases (Chung et al., 2010a; Qin et al., 2011; Zhong et al., 2011; Li et al., 2013a) (Figure 1). These results are further supported by the results from in rodent models of obstructive and remnant kidney diseases induced in mice lacking Smad3, Smad7, or having conditional knockout (KO) for Smad2 or overexpressing renal Smad7 (Chung et al., 2010a, 2013a; Qin et al., 2011; Zhong et al., 2011). This notion is firstly supported by the *in vitro* studies that TGF-βinhibits miR-29 expression but upregulates the expression of miR-21, -192, and -433 *via* the Smad3-dependent mechanism as revealed in MCs and TECs overexpressing Smad7, or knocking down for Smad2 or Smad3 and in Smad2 or Smad3 KO mouse embryonic fibroblasts (MEF) (Chung et al., 2010a, 2013a; Qin et al., 2011; Zhong et al., 2011). In addition, we also find that Smad3 physically interacts with Smad-binding site (SBE) located in their promoters to regulate the expression of these miRNAs (Chung et al., 2010a; Qin et al., 2011; Zhong et al., 2011; Li et al., 2013a). Binding of Smad3 on SBE in the promoters can either promote transcription and post-transcriptional processing of miRNAs, such as miR-21, -192, and -433, or suppress the transcription, such as miR-29b (Chung et al., 2010a; Qin et al., 2011; Zhong et al., 2011; Li et al., 2013a). Furthermore, Smad7, which is an inhibitory Smad, is able to defend kidneys from fibrosis because it can regulate TGF-β/Smad3-mediated miRNAs by maintaining renal miR-29b but inhibiting miR-21, -192, and -433 (Chung et al., 2010a, 2013a; Lan and Chung, 2012).

In addition, the feedback loop occurs as microRNAs can also regulate the TGF-β/Smad3 signaling (Figure 1). During renal injury, TGF-βinduces the miR-21 expression and this elevation of miR-21 suppresses Smad7 expression and, in turn, enhances the TGF-βsignaling (Liu et al., 2010a; Zhong et al., 2013). As a feed-forward loop, miR-21 may amplify TGF-βsignal during renal injury. MiR-21 promotes renal fibrosis may employ the activation of ERK/MAP kinase signaling in fibroblasts (Thum et al., 2008).

In contrast, TGF-βexpression can be negatively regulated by miRNAs. For instance, miR-200a is able to suppress TGF-βexpression. Although both TGF-β1 and TGF-β2 suppress miR-200a expression in renal cells (Wang et al., 2011), TGF-β2 is also one of the target genes for miR-200a. Overexpression of miR-200a inhibits TGF-β2 expression, Smad3 activity, and TGF-β1-induced fibrosis (Wang et al., 2011). These results reveal a possible feedback between TGF-β2 and miR-200a. Similarly, miR-29 is shown to inhibit TGF-β1 and TGF-β2 (van Rooij et al., 2008; Zhang et al., 2014). Thus, miR-29 may exert its anti-fibrotic effects through inhibition of TGF-βsignaling. In addition, recent studies also demonstate that TGF-β1 can be extensively post-transcriptionally regulated by miR-744 and miR-663 (Tili et al., 2010; Martin et al., 2011).

In addition, polyamine depletion activates TGF-βsignaling (Patel et al., 1998; Rao et al., 2000; Liu et al., 2003) and our laboratory recently demonstrated that depletion of cellular polyamine levels by targeting Azin1 with a Smad3-dependent miR-433 exaggerates TGF-β-induced renal fibrosis (Li et al., 2013a). Both *in vitro* and *in vivo* studies show that elevated expression of TGF-β1, TβRI, miR-433, and phosphorylated Smad3 after TGF-β1 treatment is inhibited by overexpressing Azin1 or knocking down miR-433. This inhibition is accompanied by restoring cellular polyamine levels. These results support our hypothesis that TGF-β/Smad3-miR433 signaling mediates renal fibrosis. Other studies demonstrates that the miRNAs also regulate TGF-βsignaling during renal fibrosis. For instance, TGF-βreceptor 1 (TGF-βR1) is found to be identified as a target of miR-130b but during fibrosis, miR-130b is down-regulated by TGF-β1 (Castro et al., 2014). All these results demonstrate the close relationship and the complexity between miRNAs and TGF-β-induced renal fibrosis.

## Clinical application of microRNAs in kidney diseases

### Biomarkers

The presence of miRNAs in blood and urine suggests the potential of miRNAs to be biomarkers of kidney diseases. Recently, more and more investigations imply the circulating miRNAs are potential biomarkers for cancer growth and organ injuries because miRNAs are stable and tissue specific as well as they can be identified and quantitated (Velu et al., 2012). The potential of miRNAs to be biomarkers for kidney diseases has been investigated recently. For example, remarkably high levels of circulating miR-21 is found in patients with severe interstitial fibrosis and tubular atrophy (Glowacki et al., 2013). An another study also shows that a total of 27 microRNAs at significantly different levels are found in urine from the patients at different stages of diabetic nephropathy (Argyropoulos et al., 2013). Furthermore, these 27 miRNAs have previously been found to participate into in signaling pathways of renal fibrosis during diabetic kidney disease. In addition, urinary levels of miR-29b and -29c are related to proteinuria and renal function in immunoglobulin A (IgA) nephropathy while miR-93 levels in urine are closely correlated with glomerular scarring (Wang et al., 2012). Now it should be time to search for patterns of these miRNAs that are released into the blood or urine from by diseased kidneys.

### Therapeutic potential

MiRNAs should have therapeutic potential of miRNAs in kidney diseases because miRNAs play an essential roles in renal injury. Furthermore, sequence complementarity between mRNA and miRNA offers a feasible and specific approach to develop a miRNA drug that specifically targets gene(s) or miRNA(s) which have pathologic effect on certain disease. Recent improvements in chemical engineering enable us to develop chemical modified miRNAs that are stable in the circulation, can freely move into cells to target specific mRNA or miRNA and silence it (Lorenzen et al., 2011). Conventional construction of overexpression or shRNA plasmids provides an alternative to restore or suppress miRNA transcription, respectively (Qin et al., 2011; Zhong et al., 2011). In mouse models of kidney disease, restoration of miR-29 and -200 families, or inhibition of miR-21, -192, and -433 inhibits renal fibrosis (Oba et al., 2010; Qin et al., 2011; Zhong et al., 2011, 2013; Putta et al., 2012; Chung et al., 2013a). Thus, application of miRNAs or their inhibitors provides a novel and effective therapeutic approach to the treatment of kidney diseases.

The delivery method and safety are the main concern in the therapeutic application of miRNAs. So far, systemic delivery of chemical-engineered oligonucleotides is the most method to inhibit miRNA function (Lorenzen et al., 2011). One of the possible drawback it that this method may also suppress the function of miRNAs in organs other than the diseased one. To overcome it, specific gene delivery system to limit miRNA expression in specific organs is also developing (Lan et al., 2003; Xiao et al., 2012). For example, ultrasound-microbubble-mediated gene transfer developed has been shown to be able to deliver miRNA overexpression or knockdown plasmids specifically into the living kidneys (Lan et al., 2003; Qin et al., 2011; Zhong et al., 2011, 2013; Chung et al., 2013a). For the success gene therapy, it is also essential to control the transgene expression at the desired therapeutic levels to minimize the side-effect. To achieve this, an optimal dosage of miRNAs should be considered and investigated to avoid any undesirable side-effects caused by over doses of overexpression or inhibition of miRNA expression.

The risks of off-target effects and non-specific immune response are also the main concern in miRNA therapy. For instance, the one of main considerations to apply miR-21 as a therapeutic agent for fibrotic diseases is that the inhibition of miR-21 expression will result in an induction of apoptosis (Li et al., 2009; Godwin et al., 2010; Zhong et al., 2011). Similarly, strong pro-apoptotic effect of miR-29b may also impede the development of miR-29b gene therapy as overexpression of microRNA-29b upregulate cell death of multiple myeloma cells (Zhang et al., 2011). Therefore, miRNA therapeutics still await for further improvement on the controllable delivery system specific for cells and organs.

## Summary and perspectives

After the discovery of miRNAs and characterization of their functions in kidney diseases in last two decades, it is still plenty of room for improving our understanding about the specific role and mechanism of miRNAs in renal pathophysiology. How to accurately identify miRNA targets becomes one of the key issues which hinder our progress of miRNAs in renal research because the short seed sequence of a miRNA allow it to regulate multiple target genes. Although target prediction programs provide us a large number of potential miRNA targets, the overlap among various algorithms is so minimal that only a small portion of these targets can be validated experimentally. Even after the conservation of the 3′ UTR among species is included in the investigation, the number of targets predicted is far more than those for validation. In addition, the power of miRNAs also relies on their capability of targeting multiple genes that contribute to a pathway or phenotype. However, this also introduces the difficulty to search for real targets of miRNAs. We hope that the advances in the high-throughput validation and proteomic analysis will provide a solution to identify miRNA targets.

Another difficulty of miRNA research is to understanding the regulation of miRNA expression because possibly more than one mediators or pathways participate in regulating miRNA expression. For example, within a given miRNA cluster, miRNAs may show the same pattern of expression but some of cluster members may provide different expression patterns if they do not follow the pattern (Khella et al., 2012b). Furthermore, it is common that some miRNAs may be encoded from more than one genomic loci, such as miR-29b, with very different promoter contexts (Kriegel et al., 2012b). How to control the miR-29b expression from 2 genomic loci still awaits for further investigation. In addition, intronic miRNAs provides an interesting question as they do not always have the same expression pattern as their host gene (Baskerville and Bartel, 2005). Furthermore, it is found that both strands of the miRNA are sometimes coexpressed and they usually target different sets of genes (Khella et al., 2012a). All these unsolved questions are required to have further studies to understand the mechanism about how to regulate miRNA expression. Deep sequencing is becoming a valuable tool to provide us a comprehensive view of gene expression patterns and quantification of transcript levels. This information may assist us to correlate the expression of miRNAs with the target transcripts.

Finally, microRNAs are vital downstream effectors of TGF-β-induced renal fibrosis. The further understanding of the role and mechanism of miRNAs during TGF-β-induced renal fibrosis should provide us a novel and effective strategy to halt disease progression.

### Conflict of interest statement

The authors declare that the research was conducted in the absence of any commercial or financial relationships that could be construed as a potential conflict of interest.
